# Towards malaria elimination - a new thematic series

**DOI:** 10.1186/1475-2875-9-24

**Published:** 2010-01-20

**Authors:** Marcel Tanner, Marcel Hommel

**Affiliations:** 1 Guest Editor 'Towards Malaria Elimination'; 2 Editor-in-chief, Malaria Journal

## Abstract

The launch of a new thematic series of *Malaria Journal *-- "Towards malaria elimination" -- creates the forum that allows carrying scientific evidence on how to achieve malaria elimination in specific endemic settings and conditions into the circles of scientists, public health specialists, national and global programme managers, funders and decision makers.

## 

The paradigm shift from malaria control to malaria eradication following declarations at the Gates Malaria Forum in October 2007 [1,2] and subsequent support voiced by World Health Organization (WHO) [2], the Board of the Roll Back Malaria (RBM) Partnership and many other institutions has renewed inspiration for innovation and public health action. New initiatives such as attempts to eliminate malaria in the Southern African region [3] and Pacific Island states [4] and the new global agenda and field manual for malaria elimination from WHO's Global Malaria Programme [5,6] foreshadowed this movement and are preparing the ground for another global attempt at eradication. Very swiftly a coherent global action plan for malaria eradication was established and approved by RBM in late 2008 [7]. A group of scientists, public health decision makers, control programme managers and funders, the Malaria Elimination Group, compiled - based on all currently available scientific evidence and case studies - a guide to policy makers for malaria elimination for areas that embark or have embarked on elimination strategies [8]. All these recent efforts illuminate a pathway from control through to elimination and, eventually to eradication, as the only ethical long-term strategy.

Alongside and interrelated with these important developments over the past decade, a remarkable decline of malaria incidence in several countries in sub-Saharan Africa, and world-wide, has been observed in recent years. This fall seems to have started before the widespread introduction of insecticide-treated nets and is a reflection of the renewed efforts in malaria control [9-15]. In the world today, 108 countries are malaria-free and the remaining one hundred countries still experience malaria transmission; 39 of these countries have already embarked on elimination while the remaining 61 countries implement control strategies [8].

It is against this background that the malaria community has to prepare for an "elimination/eradication era". The challenges remain formidable, but efforts must focus at all levels from developing better tools to how existing and future tools can be strategically combined for maximum synergistic effectiveness when integrated into different health and social systems prevailing in endemic areas. Any effort towards elimination - and here again one can learn from the past - needs to be (i) a synchronous global effort, locally adapted in all endemic areas, (ii) sustained over the long-term and - equally important - (iii) continuously strengthened by more basic and applied research.

Although *Malaria Journal *has already, over the past few years, published many important papers related to malaria control, it was felt that a specific, on-going, thematic series on elimination would further stimulate the debate. "Towards malaria elimination" offers the first and continuous, scientific platform on elimination and eradication. It is aimed at exchanging, discussing and developing evidence, innovation and concrete experience from all areas and levels.

For its launch, the thematic series includes two papers, one describing the assessment of parasite burden in Sri Lanka [16], the other one describing an integrated programme to eliminate malaria from the island of Pr**i**ncipe [17].

"Towards malaria elimination" welcomes original papers, reviews, commentaries and opinion pieces on malaria elimination and eradication, and hopes that this mix of scientific information and debate will catalyze and dynamize the move towards more intensified control and progressively malaria elimination within a spirit of mutual learning for change. The Editors very much hope that many scientists, public health professionals and policy makers will start using "Towards malaria elimination" as one of their ways to share new evidence or to discuss evidence presented in the scientific literature.
